# Identification and validation of a prognostic four-genes signature for hepatocellular carcinoma: integrated ceRNA network analysis

**DOI:** 10.1007/s12072-019-09962-3

**Published:** 2019-07-18

**Authors:** Yongcong Yan, Yingjuan Lu, Kai Mao, Mengyu Zhang, Haohan Liu, Qianlei Zhou, Jianhong Lin, Jianlong Zhang, Jie Wang, Zhiyu Xiao

**Affiliations:** 1 Guangdong Provincial Key Laboratory of Malignant Tumor Epigenetics and Gene Regulation, Sun Yat-Sen Memorial Hospital, Sun Yat-Sen University, Guangzhou, 510120 China; 2 Department of Hepatobiliary Surgery, Sun Yat-Sen Memorial Hospital, Sun Yat-Sen University, Yanjiang West Road 107#, Guangzhou, 510120 China; 3 Department of Oral and Maxillofacial Surgery, Sun Yat-Sen Memorial Hospital, Sun Yat-Sen University, Guangzhou, 510120 China; 4 RNA Biomedical Institute, Sun Yat-Sen Memorial Hospital, Sun Yat-Sen University, Guangzhou, 510120 China; 5 Department of Gastroenterology and Hepatology, The First Affiliated Hospital, Sun Yat-Sen University, Guangzhou, 510120 China

**Keywords:** Hepatocellular carcinoma, Overall survival, Competing endogenous RNA, Least absolute shrinkage and selection operator, Global transcriptome

## Abstract

**Background:**

Hepatocellular carcinoma (HCC) is one of the most aggressive malignant tumors, with a poor long-term prognosis worldwide. The functional deregulations of global transcriptome were associated with the genesis and development of HCC, but lacks systematic research and validation.

**Methods:**

A total of 519 postoperative HCC patients were included. We built an interactive and visual competing endogenous RNA network. The prognostic signature was established with the least absolute shrinkage and selection operator algorithm. Multivariate Cox regression analysis was used to screen for independent prognostic factors for HCC overall survival.

**Results:**

In the training set, we identified a four-gene signature (PBK, CBX2, CLSPN, and CPEB3) and effectively predicted the overall survival. The survival times of patients in the high-score group were worse than those in the low-score group (*p* = 0.0004), and death was also more likely in the high-score group (HR 2.444, *p* < 0.001). The results were validated in internal validation set (*p* = 0.0057) and two external validation cohorts (HR 2.467 and 2.6). The signature (AUCs of 1, 2, 3 years were 0.716, 0.726, 0.714, respectively) showed high prognostic accuracy in the complete TCGA cohort.

**Conclusions:**

In conclusion, we successfully built a more extensive ceRNA network for HCC and then identified a four-gene-based signature, enabling prediction of the overall survival of patients with HCC.

**Electronic supplementary material:**

The online version of this article (10.1007/s12072-019-09962-3) contains supplementary material, which is available to authorized users.

## Introduction

According to the 2018 global cancer statistics, there are 841,080 new liver cancer cases and more than 780 thousand deaths per year worldwide, and China accounts for nearly half of the total number of cases and deaths [1, 2]. Approximately 70–90% of all primary liver cancers are hepatocellular carcinoma (HCC) [3, 4].

The treatment of HCC has made encouraging progress over the past few decades and primarily consists of surgical resection, chemotherapy, molecular targeting treatment, and liver transplantation [5]. However, surgery remains the most effective treatment; it has markedly improved the overall survival (OS) of HCC patients, although the long-term survival rate is still low. Approximately 60% of patients experience recurrence or distant metastasis within 5 years [3]. Regarding the poor prognosis, many experts have identified several prognostic factors, including patient basic features (e.g., age and gender) and tumor-related factors (e.g., tumor grade), that can be used to predict the OS of HCC patients who have undergone surgery [6, 7]. However, effective prognostic factors are still lacking.

Although several studies have highlighted valuable biomarkers, these studies had limitations, including their inclusion of single-center cohorts, small populations, and single molecular markers. More importantly, most studies failed to validate their findings via another independent cohort, meaning that the results could not be generalized. Thus, few biomarkers have been utilized in clinical practice.

The competing endogenous RNA (ceRNA) hypothesis describes a novel regulatory mechanism by which mRNAs and long noncoding RNAs talk to each other using microRNA response elements (MREs) as letters to form a regulatory network across the whole transcriptome, which plays a significant role in cancer research [8, 9], such as in oral carcinoma [10] and cholangiocarcinoma [11]. Accordingly, there is a great need to explore the regulatory relationships between lncRNAs-miRNAs-mRNAs during HCC initiation and progression. Wang et al. identified a prognostic signature based on the expression profiles of six genes for the OS of HCC patients based on independent screening of Cox-penalized regressions [12]. To the best of the authors’ knowledge, there is still no report of the involvement of lncRNAs in the transcriptional regulation of miRNAs and mRNAs in the field of HCC with large-scale, high-throughput sequencing data.

In our study, we obtained lncRNA, mRNA and miRNA expression profiles and constructed the ceRNA network in HCC from the TCGA database. We identified 20 DEmRNAs involved in the ceRNA network that alone predicted the OS of HCC patients, termed “OS-genes”. Importantly, we conducted an integrated analysis of OS-genes using the logistic least absolute shrinkage and selection operator (LASSO) penalized regression to generate a four-gene-based signature (PBK, CBX2, CLSPN, and CPEB3) associated with OS in HCC. Then, we validated this signature using the internal set and two external validation cohorts, analyzed it in subgroups of HCC patients, and showed that it was an independent indicator. Thus, we identified and validated a new candidate marker to predict HCC OS by classifying patients into low- and high-risk groups.

## Materials and methods

### Patients and data collection

We downloaded level 3 data, which contained the high-throughput sequencing data of mRNAs, lncRNAs and miRNAs of 374 HCC samples and 50 normal samples from the Cancer Genome Atlas (TCGA, https://portal.gdc.cancer.gov/). Clinical data, such as prognosis and basic clinical information, were downloaded from the Data Coordinating Center (Supplementary Table S1).

The HCC patients were randomly assigned to a training set with *N* × *q* samples and an internal validation set with *N* × (1 − q) samples (*q* = 2/3). To validate our results responsibly, we searched for external validation cohorts from two independent centers. External validation cohort 1, GSE76427 (*n* = 115), microarray data and patient clinical information were downloaded from the Gene Expression Omnibus database (GEO; https://www.ncbi.nlm.nih.gov/geo/). In addition, we used another external validation cohort obtained from Sun Yat-sen Memorial Hospital between January 1, 2010, and June 30, 2010, that included 50 postoperative HCC patients (the SYMH cohort or the qPCR validation cohort). All the clinicopathological features of external validation cohorts were presented in Supplementary Table S1. All diagnoses were confirmed by pathology. This retrospective analysis was approved by the institutional review board of Sun Yat-Sen Memorial Hospital, Sun Yat-Sen University.

### Identification of DEGs

DEGs, including differentially expressed mRNAs, lncRNAs and miRNAs (DEmRNAs, DElncRNAs, and DEmiRNAs), were identified among the 354 tumor tissues and 50 normal samples. The RNA expression data from TCGA were normalized. We conducted gene identification using the edgeR package in software R, which is publicly available through Bioconductor (http://www.bioconductor.org/) [13]. |log2 fold-change| ≥ 2 and *p* value < 0.05 were used for selecting DEmRNAs and DElncRNAs. We defined and annotated DElncRNAs using the Encyclopedia of DNA Elements (ENCODE); for DEmiRNAs, the select indicator was |log2 fold-change| ≥ 1.5 and the *p* value was < 0.05.

### Seed match analysis and constructing the ceRNA network

The target mRNAs of DEmiRNAs were predicted by combined utilization in Targetscan database (http://www.targetscan.org/), miRDB database (http://www.mirdb.org/) and miRTarBase database (http://mirtarbase.mbc.nctu.edu.tw/). Then, we obtained the intersection elements between the target mRNAs and DEmRNAs, termed DEmiRNA-targeted DEmRNAs. We predicted the DElncRNAs targeted by DEmiRNAs in miRcode (http://www.mircode.org/). Cytoscape v3.5.0 software was used to build an interactive and visual ceRNA network using the Cytoscape user manual [14, 15].

### Functional enrichment analysis, gene expression correlation analysis and survival analysis

We further studied the DEmRNAs using the ceRNA network, and we conducted functional enrichment analysis using the Database for Annotation, Visualization, and Integrated Discovery (DAVID) [16]. GO biological functions and KEGG pathways were chosen with an enrichment score > 1.5 as well as a significance level of *p* < 0.05. Then, we plotted the DERNA survival curves; these curves were called OS-genes, OS-lncRNAs and OS-miRNAs, respectively. In addition, 20 OS-gene expression correlations were assessed with the Pearson correlation indicator.

### Prognostic signature screening and generation

To elucidate significant values for the 20 OS-genes, we used the logistic LASSO algorithm to select candidate OS-gene combinations that were reliably associated with HCC OS in the TCGA training set. LASSO allows the tuning of 25 parameters by fold cross-validation [17]. The risk score (RS) was calculated using the sum of the screened OS-gene expression values weighted by the coefficients from the LASSO regression model. We calculated the prognostic RS for each patient according to the following formula: RS = expression_gene1_ × β_gene1_ + ··· + expression_genen_ × β_genen_ (β: the regression coefficient derived from LASSO penalized regression) [18, 19].

### Prognostic signature validation and evaluation

To validate the robustness of the prognostic signature, we generated the RS for each patient in the TCGA internal validation set and two external validation cohorts. We defined the median RS as the cutoff point, and HCC patients were divided into low- and high-risk groups. In addition, we used the univariate and multivariate Cox regression analyses to evaluate the prognostic impact of clinicopathological features on OS. We calculated concordance indexes (c-indexes, also called HARRELL C-index), respectively. The c-index quantified the discrimination between two random patients, with a c-index of 0.5 indicating no discrimination and 1 indicating perfect discrimination. A time-dependent receiver operating characteristic (ROC) curve analysis, with 1, 2, 3, and 5 years as the cutoff values of time, was also performed to compare the true positive and true negative rates of the OS prediction [20].

### RNA extraction and real-time quantitative PCR

Fifty pairs of frozen HCC tissue and adjacent normal tissue were obtained from Sun Yat-sen Memorial Hospital. Total RNA was extracted using TRIzol reagent (Takara, Dalian, China). Reverse transcription was performed using PrimeScript RTase (Takara). The gene expression level was determined with qPCR with the help of Premix Ex Taq (Takara) and was normalized to GAPDH expression levels. We used the 2-ΔCT method to calculate expression levels. The primers were listed in Supplementary Table S2.

### Statistical analysis

Univariate and multivariate Cox regressions were performed in IBM SPSS Statistics Version 24, which was also used to generate hazard ratios (HRs) and 95% confidence intervals (CIs). Kaplan–Meier survival curves were used to estimate OS in different groups, and the survival differences were assessed by a two-sided log-rank test in GraphPad Prism 5.0. LASSO penalized regression, and ROC curve analyses were conducted in software R version 3.3.4 with relevant packages, such as package survivalROC, gplots, and glmnet. All statistical tests were two-sided, and a *p* value < 0.05 was considered statistically significant.

## Results

### Study flowchart and clinical characteristics of patients

**Fig. 1:**
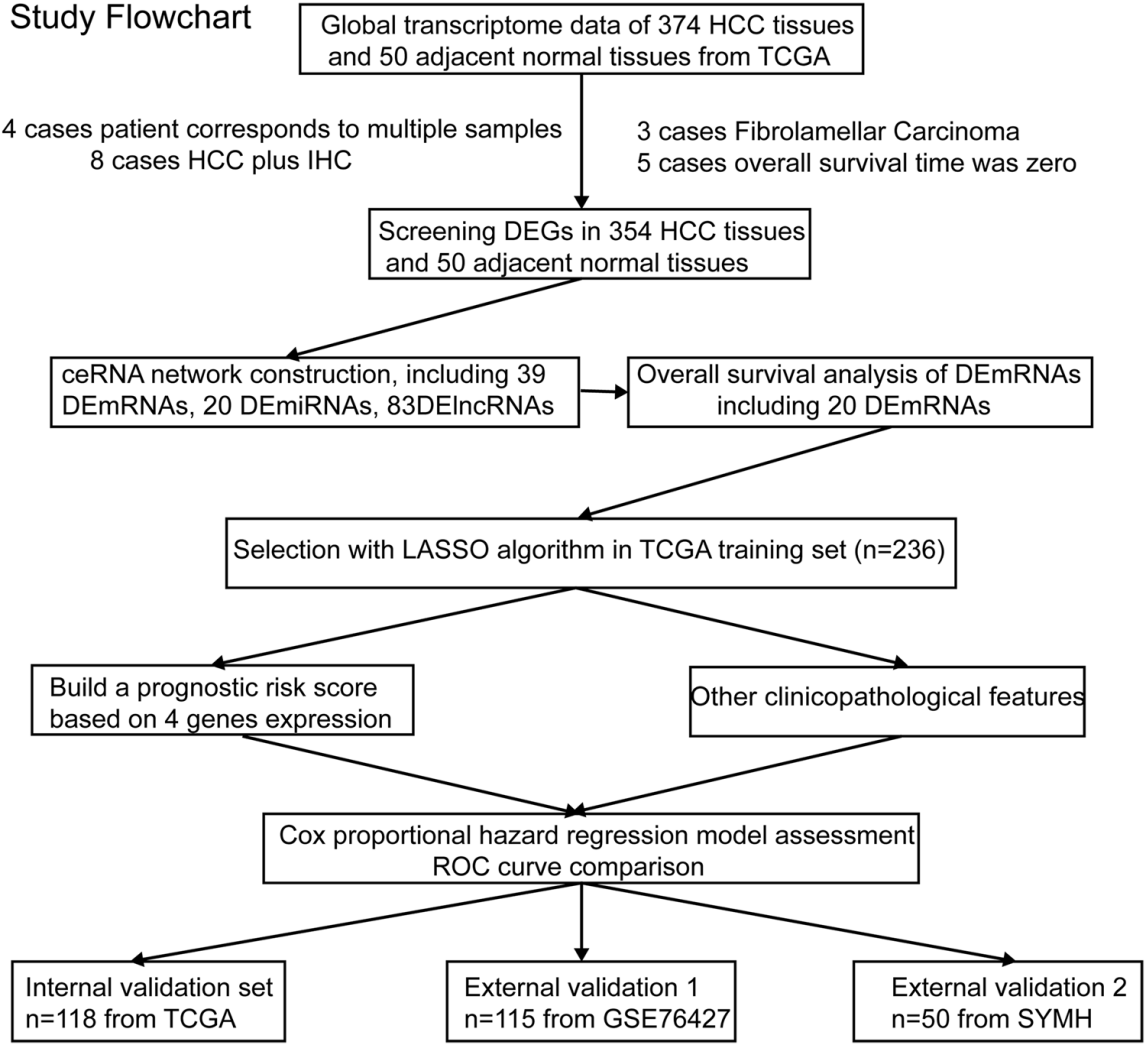
Study flowchart. *DEGs* differentially expressed genes, *LASSO* least absolute shrinkage and selector operation, *SYMH* Sun Yat-sen Memorial Hospital of Sun Yat-sen University

The study flowchart is presented in Fig. 1. A total of 354 HCC samples were included and were randomly divided into a training set (*n* = 236) and an internal validation set (*n* = 118). The median OS times of the patients in the TCGA training set, the TCGA validation set, the entire TCGA cohort, the GSE76427 cohort, and the SYMH cohort were 1694 (1068–2320), 1852 (883–2821), 1694 (1203–2185), 2296 (1534–3057) and 768 (554–981) days, respectively.

### Differentially expressed genes and construction of the ceRNA network

A total of 1993 DEmRNAs were identified, including 1788 (89.71%) that were upregulated and 205 (10.29%) that were downregulated. In addition, we found 1071 differentially expressed lncRNAs, including 1014 (94.67%) upregulated and 57 (5.32%) downregulated DElncRNAs. However, we found only 162 (95.29%) upregulated and 8 (4.71%) downregulated DEmiRNAs. We generated a heat map and volcano with complete linkage clustering of DEmRNAs, DElncRNAs, and DEmiRNAs (Supplementary Figure S1).

**Fig. 2:**
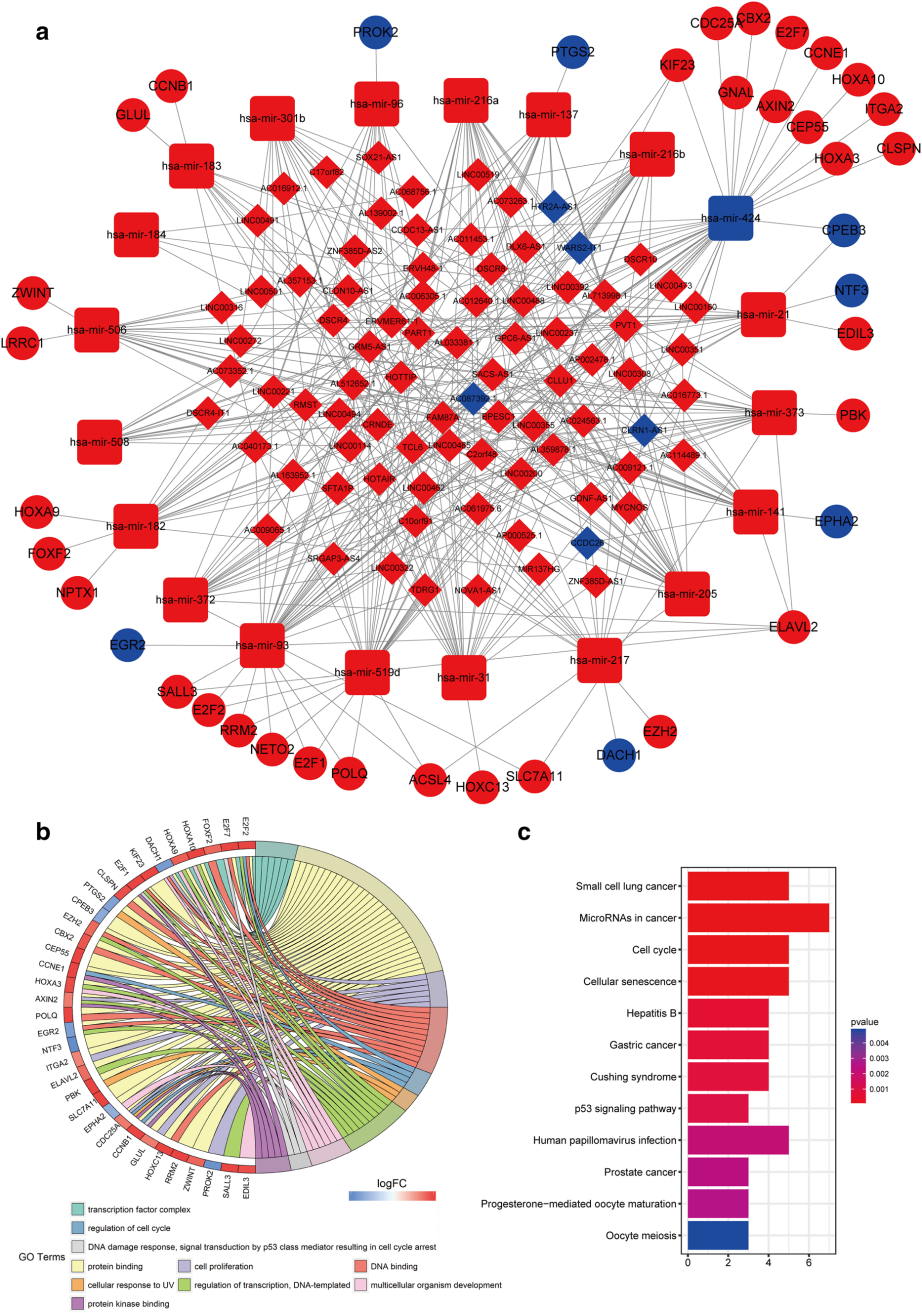
The ceRNA network of lncRNAs–miRNAs–mRNAs and functional analysis for DEmRNAs in HCC. To better understand how mRNA expression was regulated by lncRNA through combining miRNAs, we built a ceRNA visual network including 39 DEmRNAs, 83 DElncRNAs, and 20 DEmiRNAs from the TCGA database (**a**). Red represents upregulated DEGs, and blue represents downregulated DEGs. Foursquares: miRNAs, balls: mRNAs, diamonds: lncRNAs. To better elucidate the underlying pathways and biological mechanisms involved in the ceRNA network, we conducted GO (**b**) and KEGG pathway analyses (**c**) using the DAVID database for 39 DEmRNAs. *DEmRNAs* differentially expressed mRNAs

To better understand how mRNA expression was regulated by lncRNA through combining miRNAs, we built a ceRNA visual network (Fig. 2a). According to seed match analysis, we found that 39 DEmRNAs were targets of the 20 DEmiRNAs, while 83 DElncRNAs interacted with the 20 DEmiRNAs (Supplementary Tables S3 and S4).

### Functional enrichment and survival analyses of key ceRNAs

We conducted GO and KEGG pathway analyses using the DAVID database for 39 DEmRNAs (Fig. 2b, c). Many cancer-related GO items and KEGG pathways were significantly enriched, such as those associated with biological processes (e.g., cell proliferation and cell cycle) and pathways (e.g., hepatitis B and the p53 signaling pathway). Twenty mRNAs (OS-genes) (Supplementary Figure S2), one miRNA (miR-137), and 14 lncRNAs (OS-lncRNAs) (Supplementary Figures S3) were found to be significantly associated with OS. There was coexpression between 20 OS-genes; for example, the coexpression coefficient between CPEB3 and CCNB1 was − 0.57 (*p* < 0.001), whereas the coexpression coefficient between PBK and CCNB1 was 0.8 (Supplementary Figures S4I). In the future, the potential mechanisms underlying their correlations could be investigated.

### Building a predictive signature from the TCGA training set

**Fig. 3:**
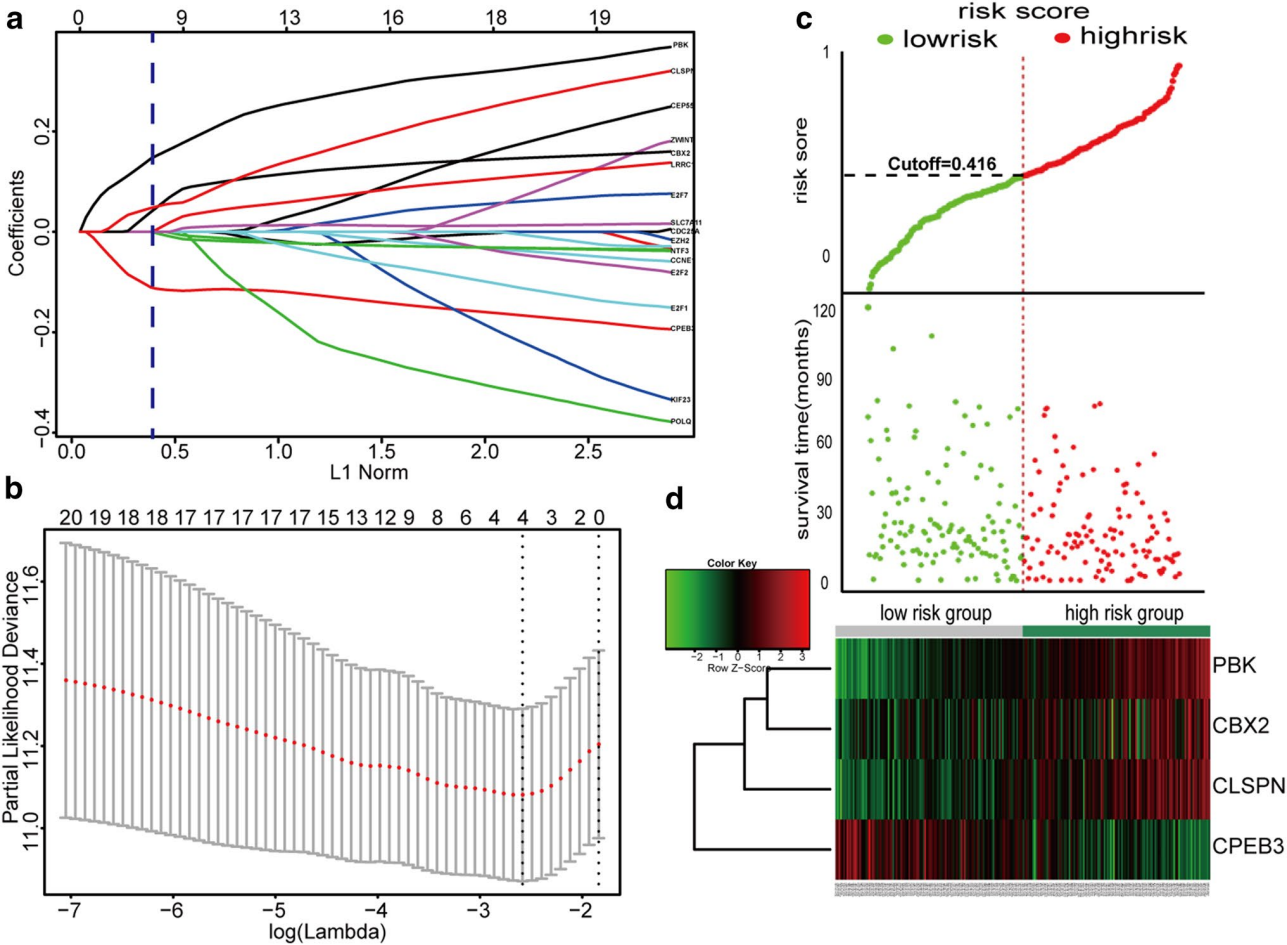
Construction of the integrated prognostic signature in the training set. **a** LASSO coefficient profiles of the 20 OS-genes. The vertical blue dotted lines are plotted at the value selected in **b**. **b** Selection of the tuning parameter (lambda) in the LASSO model by tenfold cross-validation based on minimum criteria for OS; the lower *X* axis shows log (lambda), and the upper *X* axis shows the average number of OS-genes. The *Y* axis indicates partial likelihood deviance error. Red dots represent average partial likelihood deviances for every model with a given lambda, and vertical bars indicate the upper and lower values of the partial likelihood deviance errors. The vertical black dotted lines define the optimal values of lambda, which provides the best fit. **c**, **d** Prognostic classifier analysis. **c** The RS distribution and survival time of each patient; 236 patients were divided into low- and high-risk groups according to the median RS value. **d** Heat map of the mRNAs in the prognostic signature. *RS* risk score

Twenty OS-genes were identified after we combined the DEmRNAs selected by the ceRNA network and survival analysis. We then used the LASSO regression model to further identify an optimal subset of gene-based signatures reliably associated with HCC OS in the TCGA training set. As a result, four genes were identified: PBK, CBX2, CLSPN, and CPEB3 (Fig. 3). To better clarify the performance of our predictive signature for HCC OS, we established RS with each gene coefficient weighted by the LASSO model. The RS was calculated for each patient in the training set as follows:

RS = (− 0.0922 × expression level of CPEB3) + (0.1215 × expression level of PBK) + (0.0128 × expression level of CBX2) + (0.0377 × expression level of CLSPN).

### Effective prognostic signature in HCC patients

**Fig. 4:**
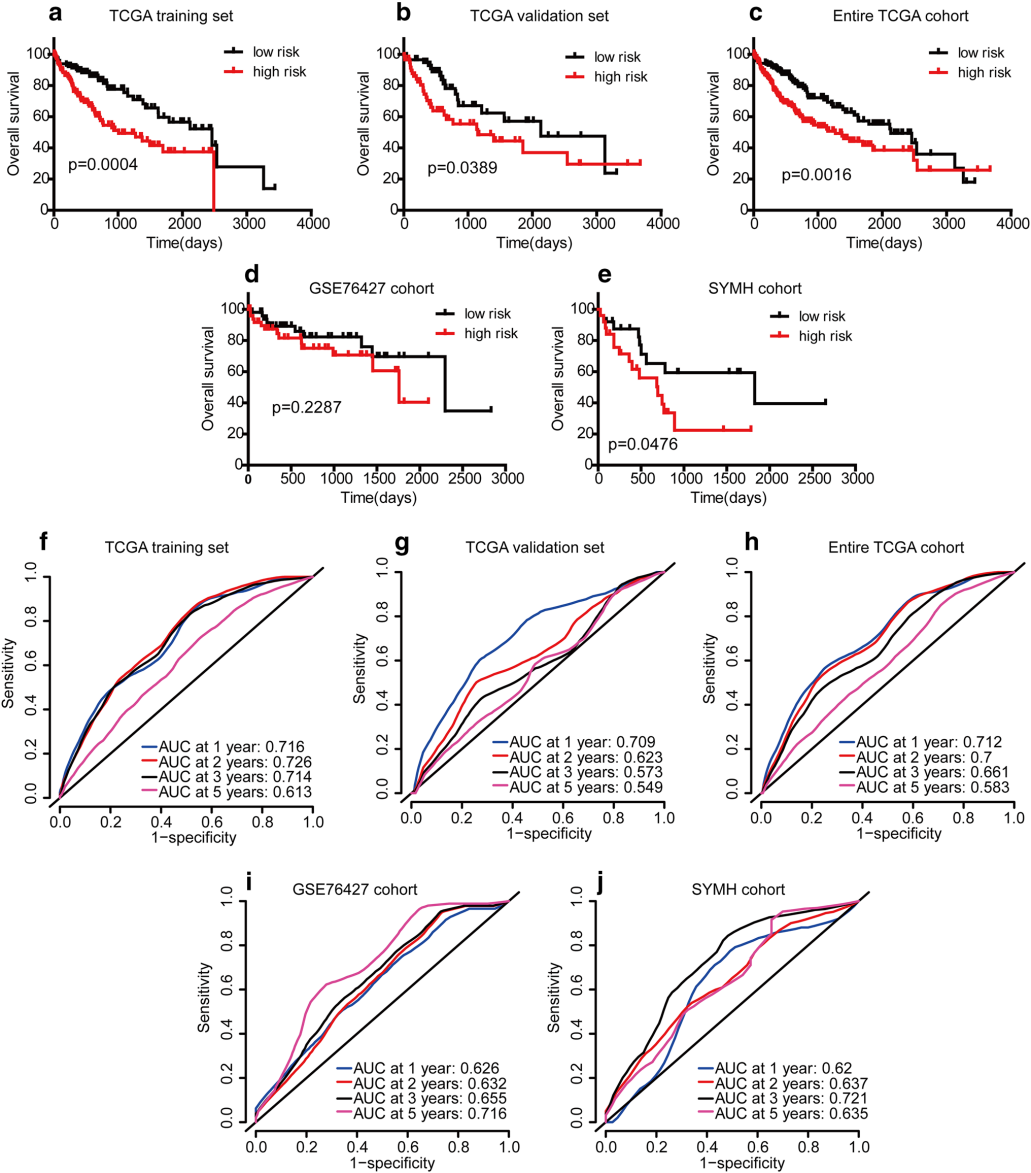
Survival analysis and ROC analysis of the four-gene-based prognostic signature in independent cohorts. Comparison of overall survival times between the low- and high-risk groups in the five data sets. Time-dependent ROC curve comparison of the five data sets; AUCs at 1, 2, 3 and 5 years were calculated. TCGA training set (**a**, **f**); TCGA validation set (**b**, **g**); entire TCGA cohort (**c**, **h**); GSE76427 cohort (**d**, **i**); SYMH cohort (**e**, **j**). *ROC* receiver operating characteristic

In the training set, 236 HCC patients were assigned to the low-score and high-score groups based on the median RS value (0.416). Patients in the high-score group exhibited worse survival than those in the low-score group as shown in Fig. 4a (*p* = 0.0004). In addition, survival analysis showed serum AFP, TNM stage, T stage, N stage, and M stage were found to be significantly associated with HCC OS (Supplementary Figure S5). We further investigated various subgroups of individual clinicopathological features in HCC patients and found that they were significantly correlated with OS because of imbalances between the high-score and low-score groups with respect to clinical features (Table 1). Subgroup analysis of the four-gene signature in the complete cohort was performed, and significant correlations between RS and OS were maintained in Asians (*p* < 0.001, Supplementary Figure S6A and S6B) and in patients whose serum AFP ≥ 20 ng/ml (*p* = 0.079, Supplementary Figures S6C and S6D), whereas RS value was associated with OS for the two subgroups of TNM stage and tumor grade (Supplementary Figures S6E-H).

**Table 1:** Relationship between four-gene signature and other clinicopathological features in TCGA cohort

Clinicopathological variables	TCGA cohort								
	Training set (*n *= 236)			Validation set (*n *= 118)			Entire cohort (*n *= 354)		
	Low risk	High risk	*P* value	Low risk	High risk	*P* value	Low risk	High risk	*P* value
Age									
< 60	41	61	0.009**	27	32	0.357	70	91	0.025*
≥ 60	77	57		32	27		107	86	
Gender									
Female	33	45	0.097	18	19	0.843	51	64	0.14
Male	85	73		41	40		126	113	
Race									
White	68	48	0.005**	31	27	0.378	98	76	0.01*
Asian	41	62		22	27		64	88	
Family History									
Negative	64	71	0.454	26	37	0.03*	92	106	0.081
Positive	35	31		27	16		62	47	
Serum AFP									
< 20 ng/ml	55	36	0.003**	33	18	0.026*	87	55	< 0.001**
≥ 20 ng/ml	33	53		17	24		49	77	
Vascular invasion									
Negative	72	57	0.104	37	32	0.864	110	88	0.086
Positive	33	42		15	12		46	56	
Fibrosis									
Negative	23	17	0.935	18	14	0.649	40	32	0.929
Fibrosis	25	20		12	10		37	30	
Cirrhosis	23	20		16	8		39	28	
TNM staging									
I–II	89	77	0.003**	47	32	0.015*	138	107	< 0.001**
III–IV	18	36		9	22		26	59	
Tumor grade									
I–II	88	62	< 0.001**	46	25	< 0.001**	137	84	< 0.001**
III–IV	27	56		13	32		37	91	
Positive	50	59		27	32		75	93	

Chi square test was used for comparison between two groups

*AFP* α-fetoprotein, *TNM* tumor-lymph node-metastasis, *OS* overall survival, *DFS* disease-free survival

*P < 0.05, **P < 0.01

### Validating and evaluating the signature

Similar analyses demonstrated that the high-score group had a worse OS than that in the low-score group in the internal validation set (median OS, 1149 days versus 2131 days; *p* = 0.0389) (Fig. 4b). For the entire TCGA cohort of 354 patients, OS for the high-score patients was shorter than for the low-score patients (median OS, 1271 days versus 2132 days; *p* = 0.0016) (Fig. 4c). The median OS times for the low-score and high-risk groups were 2296 and 1759 days, respectively, although this difference was not statistically significant in GSE76427 external validation cohort (Fig. 4d). This pattern of another external validation cohort-SYMH data set was similar to that observed in the TCGA cohort (Supplementary Figure S4A-H). Similarly, patients with a low score generally had a better OS than patients with a high score and the median OS times of the two groups were 1825 and 695 days, respectively (*p* = 0.0476, Fig. 4e).

### Cox proportional hazards regression analysis in validation cohorts

For the entire TCGA cohort, serum AFP, TNM stage, and the signature were significantly associated with HCC OS in the univariate analysis. A multivariate regression analysis indicated that TNM stage and signature were independent prognostic predictors of OS (Table 2). Furthermore, multivariate survival analysis showed that the four-gene signature could be an independent prognostic factor (HR 2.467, *p* = 0.021) in the GSE76427 cohort and was the only independent prognostic predictor of OS in the SYMH cohort (HR 2.6, *p* = 0.037) (Table 2).

**Table 2:** Univariate and multivariate Cox regression analyses of four-gene signature and other prognostic factors for OS in TCGA cohort, GSE76427 and SYMH cohort

Overall survival	Univariate analysis			Multivariate analysis		
	HR	95% CI	*P* value	HR	95% CI	*P* value
Entire TCGA cohort						
Age (≥ 60 vs < 60)	1.207	0.849–1.715	0.295			
Gender (male vs. female)	0.821	0.575–1.174	0.280			
Race (asian vs white)	0.746	0.510–1.091	0.131			
Family history (positive vs negative)	1.176	0.812–1.703	0.392			
Serum AFP (≥ 20 ng/ml vs. < 20 ng/ml)	1.656	1.064–2.578	0.025*	1.280	0.793–2.064	0.312
Vascular invasion (positive vs negative)	1.400	0.921–2.216	0.115			
Cirrhosis (fibrosis vs negative)	0.807	0.435–1.494	0.495			
(Cirrhosis vs negative)	0.753	0.404–1.402	0.371			
TNM staging (III–IV vs. I–II)	2.520	1.768–3.592	<0.001**	1.885	1.156–3.072	0.011*
Tumor grade (III–IV vs. I–II)	1.081	0.751–1.554	0.676			
Signature (high risk vs low risk)	1.753	1.231–2.497	0.002**	1.676	1.045–2.686	0.032*
GSE76427 cohort						
Age (≥ 60 vs < 60)	1.786	0.733–4.348	0.202			
Gender (male vs. female)	0.808	0.186–3.520	0.777			
TNM staging (III–IV vs. I–II)	2.340	0.977–5.603	0.056	1.897	0.607–5.932	0.271
BCLC stage (B + C vs. A)	2.508	1.070–5.879	0.034*	2.061	0.7–6.07	0.189
Signature (high risk vs low risk)	1.679	0.715–3.946	0.234	2.467	1.068–5.927	0.021*
SYMH cohort						
Age (≥ 60 vs < 60)	0.904	0.355–2.301	0.833			
Gender (male vs. female)	1.371	0.406–4.626	0.611			
Family history (positive vs negative)	1.484	0.549–4.006	0.436			
Serum AFP (≥ 20 ng/ml vs. < 20 ng/ml)	1.016	0.416–2.477	0.973	1.005	0.401–2.515	0.992
Vascular invasion (positive vs negative)	1.157	0.456–2.936	0.759			
Cirrhosis (positive vs negative)	1.264	0.565–2.828	0.569			
TNM staging (III–IV vs. I–II)	1.257	0.537–2.944	0.598	1.584	0.638–3.932	0.322
Tumor grade (III–IV vs. I–II)	0.787	0.312–1.986	0.613			
Signature (high risk vs low risk)	2.336	0.983–5.553	0.055	2.6	1.057–6.395	0.037*

*SYMH* Sun Yat-Sen Memorial Hospital, *AFP* α-fetoprotein, *TNM* tumor-lymph Node metastasis, *BCLC* Barcelona Clinic Liver Cancer, *OS* overall survival, *DFS* disease-free survival, *NA* not available, *HR* hazard ratio, *95% CI* 95% confidence interval

*P < 0.05, **P < 0.01

### Comparison with other prognostic factors

Time-dependent ROC curve analysis suggested that the four-gene signature was a stable predictor and even contained censored survival data (Fig. 4f–j).

In addition, the signature may achieve a more stable value in 2 years-OS prediction (Supplementary Tables S5). As shown in Supplementary Table S6, the signature incorporating four-genes expression achieved stable c-indexes in predicting HCC OS in the training and various validation sets (including internal and external validations).The signature was also significantly more specific and sensitive than other clinicopathological risk factors for the entire TCGA cohort and the two external validation cohorts (Supplementary Figure S7A-C). To develop a more reliable predictive model, we combined two independent prognostic factors; as a result, the four-gene signature and TNM stage (AUC 0.668, *p* < 0.05) had a more sensitive predictive value in the entire TCGA cohort (Supplementary Figure S7D). The combination of signature and TNM staging (or BCLC staging) had a higher AUC than the signature alone, although the difference was not significant for the GSE76427 cohort (Supplementary Figure S7E). However, for the SYMH cohort, there was no difference between the AUCs for the signature alone and the combination (Supplementary Figure S7F).

## Discussion

More and more evidence demonstrates that genetic alterations and disorders in the signaling pathways are of significance in tumorigenesis and the progression of HCC, meaning that molecular markers are equally important in the prediction of HCC OS. Certainly, many molecular markers have been identified to predict HCC OS. Jin et al. found that SUOX (sulfite oxidase), as an independent prognostic factor of HCC, showed better associations with OS and TTR if combined with serum AFP in different cohorts [21]. Tao et al. found that BTBD7 expression combined with microvessel density could better predict HCC prognosis by Cox regression analysis [22]. However, most of the recent research has focused on single gene expression, a specific protein, lncRNAs or miRNAs. However, information is now rapidly emerging on the vital functional role of the molecular network in HCC initiation and progression, indicating that we should analyze the prognosis markers as a whole. But sometimes we have high-dimensional data. At the time, lasso regression was the selective method for improving prediction accuracy. Lasso has two important characteristics, one is feature selection: automatic selection of features, it will learn to remove features without information and precisely set the weights of these features to zero, especially for high-dimensional data. Another one is interpretability: models are easier to explain, for example, we can find the independent variables that provide the most important information in the model when we have a lot of independent variables [23–26]. Li et al. identified 13 differentially expressed miRNAs in the serum of HER2 + MBC patients with distinct responses to trastuzumab using miRNA microarrays and constructed a four-miRNA signature to predict survival using a LASSO model [27]. Backes et al. [28] used multivariable Lasso regression to develop models to identify patients most likely to benefit from adjuvant surgery by projecting their case–control data towards the entire cohort. Transcriptome profiling revealed an integrated signature, incorporating 15 mRNAs and three lncRNAs, was a powerful predictor of early relapse and had a better OS prediction than TNM staging in colon cancer [29].

In the present study, we conducted a comprehensive analysis of whole transcriptome resequencing data and its involvement in the prediction of HCC OS. First, we identified DEGs, including a total of 1993 differentially expressed mRNAs (DEmRNAs), 1071 differentially expressed lncRNAs and 170 DEmiRNAs. After building a ceRNA visual network, we found 39 DEmRNAs, 83 DElncRNAs and 20 DEmiRNAs. Some of them were reported to be cancer-related genes, such as CCNB1 [30], EZH2 [31, 32], AXIN2, [33] and FOXF2 [34]. We also found several significant HCC-associated lncRNAs in our ceRNA network, such as HOTAIR [35, 36] and HOTTIP [37]. Interestingly, we noticed that lncRNA LINC00221 interacted with 12 miRNAs. Thus, LINC00221 may serve as a key regulator. Next, we studied its specific biological functions and regulatory mechanisms in HCC. Notably, miR-137 was associated with HCC OS, and in the network, we found that its corresponding mRNA was PTGS2, a key oncogene in HCC [38]. Its candidate corresponding lncRNAs were HOTTIP, CLLU1, and GPC6-AS1. In the future, we will conduct an in-depth study of the regulatory mechanisms underlying the miRNA137-PTGS2-lncRNA network.

Subsequently, we identified a four-gene-based signature (weighted combination of PBK, CBX2, CLSPN, and CPEB3) and effectively predicted OS in HCC patients using LASSO penalized regression. PBK (PDZ-binding kinase) phosphorylates MAPKp38 and plays a crucial role in the activation of lymphoid cells. Phosphorylated PBK interacts with TP53, leading to TP53 destabilization and decreased expression following doxorubicin-related DNA damage [39, 40]. CBX2 (Chromobox protein homolog 2) was composed of multi-protein PRC1-like complex, which inhibited the transcriptional activities of many genes, including the HOX genes [41]. Although CBX2 has been less-studied in cancer research, the molecular profile of CBX2 suggested that it plays an oncogenic role [42]. CLSPN, which monitors the integrity of DNA replication forks, was essential for checkpoint-regulated cell cycle arrest in response to UV irradiation-induced DNA damage [43]. Choi et al. reported that CLSPN positively affected the survival of cancer cells and negatively affected the metastasis model in response to radiation [44]. CPEB3 (cytoplasmic polyadenylation element-binding protein 3) contains an intron-encoded self-cleaving ribozyme that is structurally and biochemically associated with human HDV ribozymes, regulating its own translation [45]. CPEB3 suppresses Stat5b-dependent EGFR gene transcription in neurons [46]. All four genes may serve as key regulatory genes for cell behaviors and functions, but their abstract functions have not yet been elucidated in HCC. In the future, we will conduct an in-depth study of the regulatory mechanisms for four genes (PBK, CBX2, CLSPN, and CPEB3) based on their ceRNA network clarified in present study.

Although we constructed an OS-related predictive model based on OS-related data, we surprisingly found that the model may also serve as a tool to forecast disease-free survival (DFS) to some extent (data are not shown), low score represents a long DFS, while high-score means that patient may suffer a poor DFS, but more cohort studies are needed to confirm this.

OS for HCC is multifactorial and cannot be only determined by gene expression. HCC development is driven by the interaction of genetic predisposition, environmental factors (metabolic syndrome, alcohol, and aflatoxin B1) and viruses (HBV and HCV). Hepatocarcinogenesis is a multi-step process, and driving forces in hepatocyte transformation, HCC development and progression are chronic inflammation, DNA damage, epigenetic modifications, senescence and telomerase reactivation, chromosomal instability, and early neoangiogenesis [47]. In the recent years, genome-wide technologies and next-generation sequencing have enabled the identification of molecular signatures to classify subgroups of HCCs and stratify patients according to prognosis. Unraveling the patterns of genomic alterations in HCCs is pivotal towards identifying targeted therapies [48, 49]. We tried to build a model based on genomic alterations which was associated with OS, and help us better formulate individual treatment and follow-up management strategies which meet the requirements of precision medicine to a certain extent. We could imagine two HCC patients: X and Y. They have the same age, sex, and BCLC stage. However, both patients are stratified into same stage of disease, which is associated with specific outcomes. As has been widely acknowledged, the two patients will probably have different prognoses, but the question regarding how to quantify these prognoses remains unresolved. In our model, we tried to calculate the total scores of the signature individually based on molecular medicine. Different scores correspond to different prognosis. If the patients have a higher score, we would maintain closer follow-up and medical treatment.

Similar to our investigation, Wang et al. identified a prognostic signature based on the expression profiles of six genes for the OS of HCC patients, including SRL, TTC26, CPSF2, TAF3, C16orf46, and CSN1S1, based on independent screening of Cox-penalized regressions [12]. Compared with previous studies, our study has several strengths. First, we used large-scale, high-throughput sequencing data from the TCGA database, rather than that from a single medical center, to avoid heterogeneity among different centers. Second, we established a lncRNA–miRNA–mRNA ceRNA network among the DEGs in tumor tissues and normal liver tissues. Third, we performed an in-depth screening study of DEmRNAs that were not only involved in the ceRNA network but also associated with the OS of HCC patients based on LASSO regression, in contrast to previous studies that used only one method to select prognostic markers. Fourth, we conducted internal validation and independent external validations, thus rendering the results more reliable and useful.

Survival analysis showed serum AFP, TNM stage, T stage, N stage, and M stage were found to be significantly associated with HCC OS. We further investigated various subgroups of individual clinicopathological features in HCC patients and found that they were significantly correlated with OS because of imbalances between the high-score and low-score groups with respect to clinical features. Significant correlations between signature and OS were maintained in Asians and in patients whose serum AFP ≥ 20 ng/ml. The four-gene signature was an independent prognostic factor in multivariate Cox regression and subgroup analysis, particularly for Asians patients with serum AFP ≥ 20 ng/ml.

Inevitably, our study had several limitations. First, the multivariable survival analysis contained only basic prognostic factors from the GEO database and was unable to suggest other possible clinical factors, such as status of the metastatic lesions and performance status of patients. Second, as we know, extensive evidence indicates that HCC is an extremely heterogeneous tumor at the genetic and molecular level, limited by the data of the study, all genes’ expression from TCGA, GEO, and SYMH cohorts were detected in a piece of HCC tissue from one patient. In the future, we will detect the expression of the four genes by single-cell whole-genome sequencing or quantitative RT-PCR analysis in several pieces of HCC specimens from one patient, so that we can know whether the four-gene signature is a reliable and workable OS prediction marker for HCC. In addition, we will seek for cooperation with other hospital to obtain more patients and tissues for the gene model validation.

## Conclusions

We constructed a novel lncRNAs–miRNAs–mRNAs ceRNA network in HCC based on genome-wide analysis, then we identified and validated a new candidate therapeutic decision marker based on the ceRNA network that yields great promise in the prediction of HCC OS in the future.

## Electronic supplementary material

Below is the link to the electronic supplementary material.

Figure S1 Hierarchical clustering and volcano plots of DEGs. Hierarchical clustering of HCC tissues and normal live tissues by differentially expressed mRNAs (A), lncRNAs (C) and miRNAs (E). The lower horizontal axis represents samples, and the upper horizontal axis represents clusters of samples. The left vertical axis represents clusters of DEGs, and the right vertical axis represents DEG names. Red represents upregulated DEGs, and green represents downregulated DEGs. Volcano plots of differentially expressed mRNAs (B), lncRNAs (D) and miRNAs (F). The red dots represent upregulated DEGs, and the green dots represent downregulated DEGs. FDR, false discovery rate (TIFF 5915 kb)

Figure S2 Kaplan–Meier survival curves for 20 DEmRNAs (all were associated with the OS of HCC patients and were included in the ceRNA network). OS, overall survival (TIFF 4777 kb)

Figure S3 Kaplan–Meier survival curves for 14 DElncRNAs and miRNA-137 (all were associated with OS of HCC patients and involved in the ceRNA network) (TIFF 11081 kb)

Figure S4 The expression levels of the four genes in the signature and hierarchical clustering in the correlation matrix of 39 DEmRNAs involved in the ceRNA network. The expression levels of the four genes in the prognostic signature in the complete TCGA cohort and SYMH cohort. PBK (A, E), CBX2 (B, F), CLSPN (C, G), and CPEB3 (D, H). Pearson correlation analysis was used to calculate collinearity between the 20 OS-genes in the corresponding row and column (I) (TIFF 7362 kb)

Figure S5 Kaplan–Meier survival curves stratified by age (A), gender (B), race (C), family history (D), serum AFP (E), invasion (F), fibrosis (G), grade (H), TNM (I), T stage (J), N stage (K), and M stage (L) (TIFF 9125 kb)

Figure S6 Kaplan–Meier survival analysis for the low- and high-risk groups, stratified by race (A, B), serum AFP (C, D), TNM stage (E, F) and tumor grade (G, H) (TIFF 8329 kb)

Figure S7 Comparisons of the predictive values for OS of the four-gene-based signature and clinicopathological risk factors according to ROC analysis for 1 and 3 years (A–C). Comparisons of the predictive values for OS of the signature and a combination of the signature and important prognostic factors for independent cohorts (D–F). The AUC was calculated, and its 95% CI was estimated with bootstrap means. The *P* values were two-sided; HR hazard ratio; 95% CI, 95% confidence interval. (TIFF 10715 kb)

Supplementary material 8 (DOCX 26 kb)

## Abbreviations

HCC: Hepatocellular carcinoma
ceRNA: Competing endogenous RNA
TCGA: The Cancer Genome Atlas
DEGs: Differentially expressed genes
DERNA: Differentially expressed RNA
DEmRNA: Differentially expressed mRNA
DElncRNA: Differentially expressed lncRNA
DEmiRNA: Differentially expressed miRNA
LASSO: Least absolute shrinkage and selector operation
ROC: Receiver operating characteristic
OS: Overall survival
DFS: Disease-free survival
NGS: Next-generation sequencing
GEO: Gene expression omnibus
SYMH: Sun Yat-sen Memorial Hospital
GO: Gene ontology
KEGG: Kyoto encyclopedia of genes and genomes
DAVID: Database for annotation, visualization, and integrated discovery
RS: Risk score
HR: Hazard ratio
CI: Confidence interval
AFP: α-Fetoprotein
TNM: Tumor-lymph node-metastasis
BCLC: Barcelona Clinic Liver Cancer
