# Supplementary figures and images for: Identification and validation of a prognostic four-genes signature for hepatocellular carcinoma: integrated ceRNA network analysis
Source: Hepatol Int. 2019 Jul 18;13(5):618–30. doi: 10.1007/s12072-019-09962-3 (PMC6744548; doi:10.1007/s12072-019-09962-3)

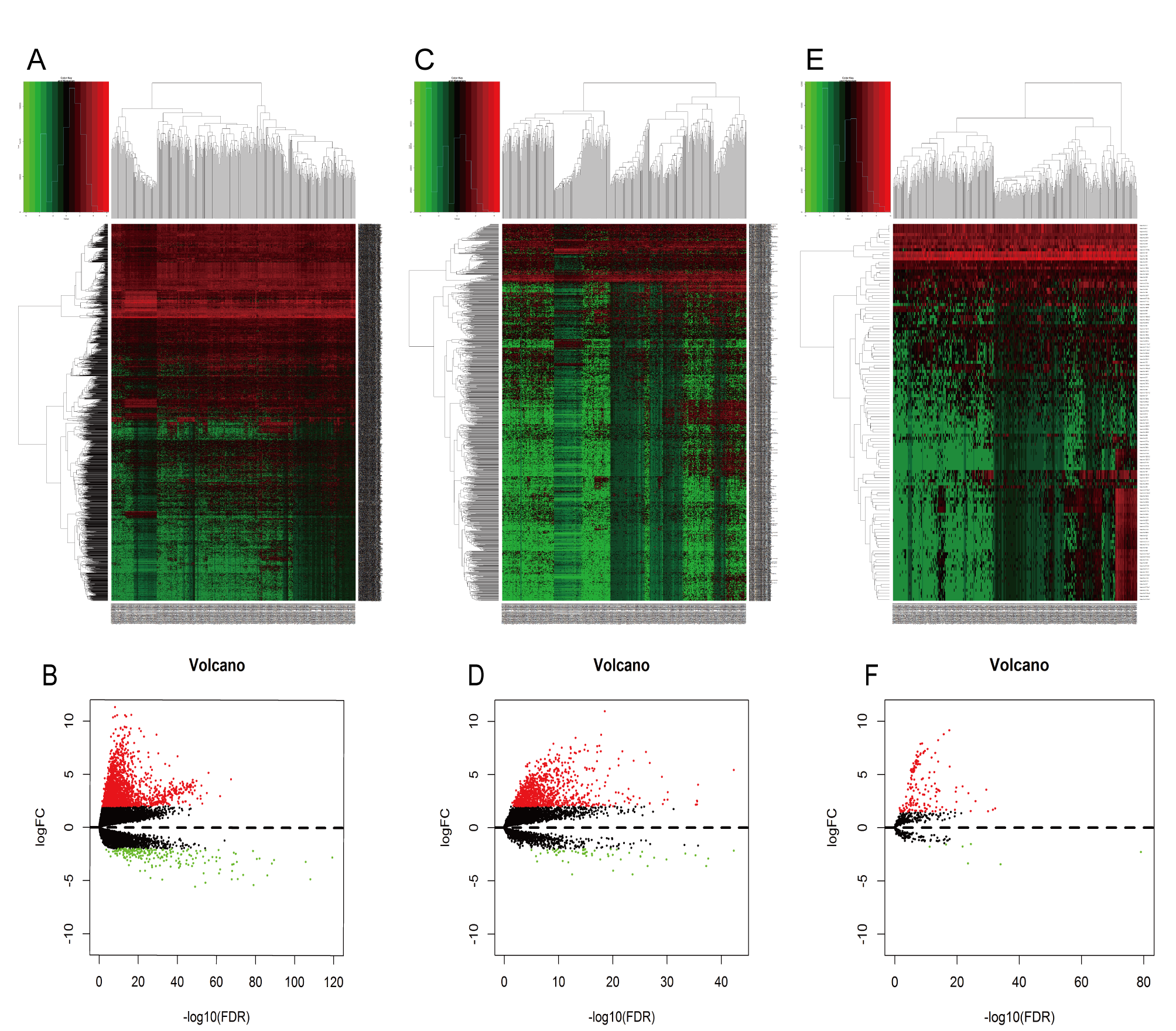

Supplement: Supplementary file 1 — Figure S1 Hierarchical clustering and volcano plots of DEGs. Hierarchical clustering of HCC tissues and normal live tissues by differentially expressed mRNAs (A), lncRNAs (C) and miRNAs (E). The lower horizontal axis represents samples, and the upper horizontal axis represents clusters of samples. The left vertical axis represents clusters of DEGs, and the right vertical axis represents DEG names. Red represents upregulated DEGs, and green represents downregulated DEGs. Volcano plots of differentially expressed mRNAs (B), lncRNAs (D) and miRNAs (F). The red dots represent upregulated DEGs, and the green dots represent downregulated DEGs. FDR, false discovery rate (TIFF 5915 kb) [file 12072_2019_9962_MOESM1_ESM.tif]

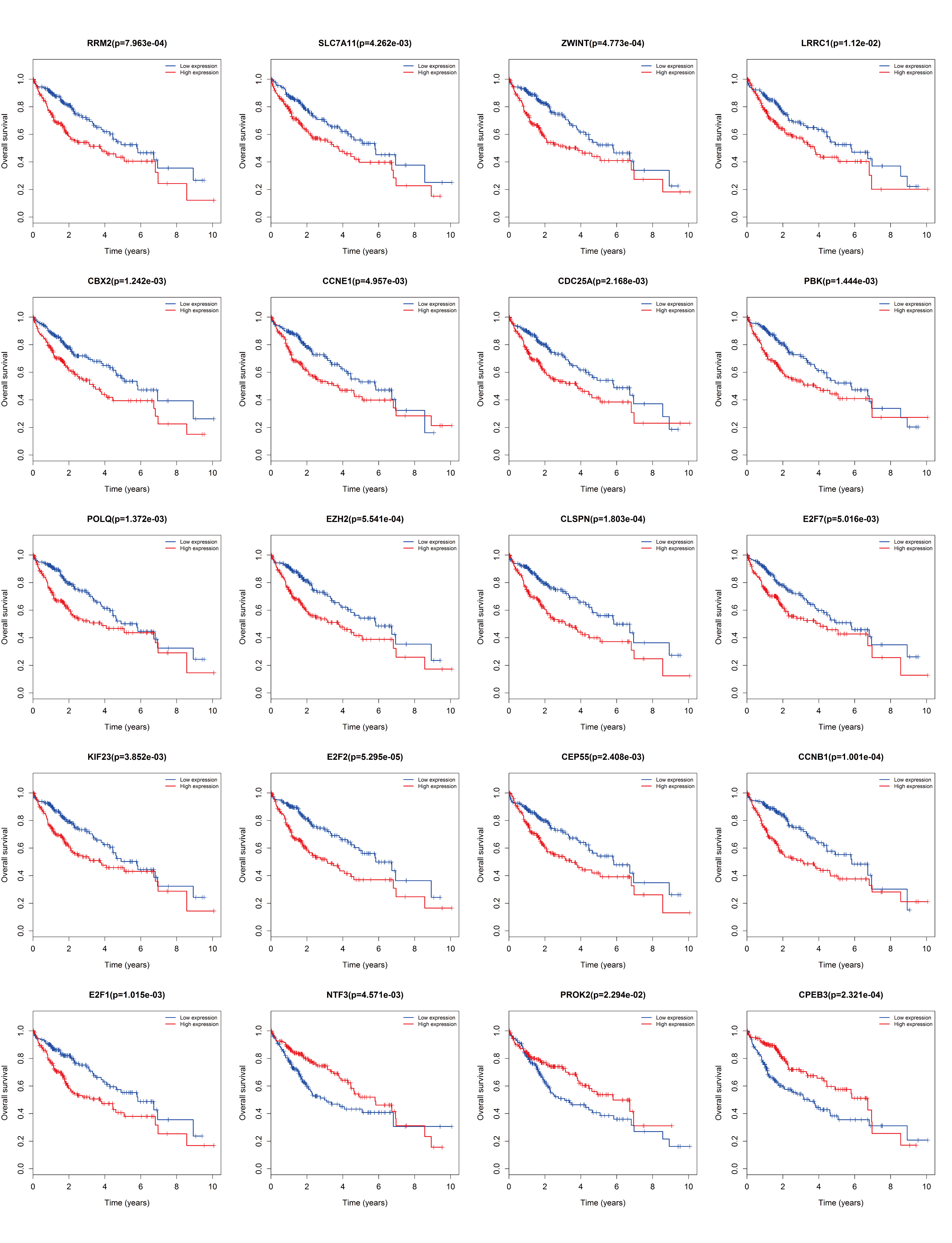

Supplement: Supplementary file 2 — Figure S2 Kaplan–Meier survival curves for 20 DEmRNAs (all were associated with the OS of HCC patients and were included in the ceRNA network). OS, overall survival (TIFF 4777 kb) [file 12072_2019_9962_MOESM2_ESM.tif]

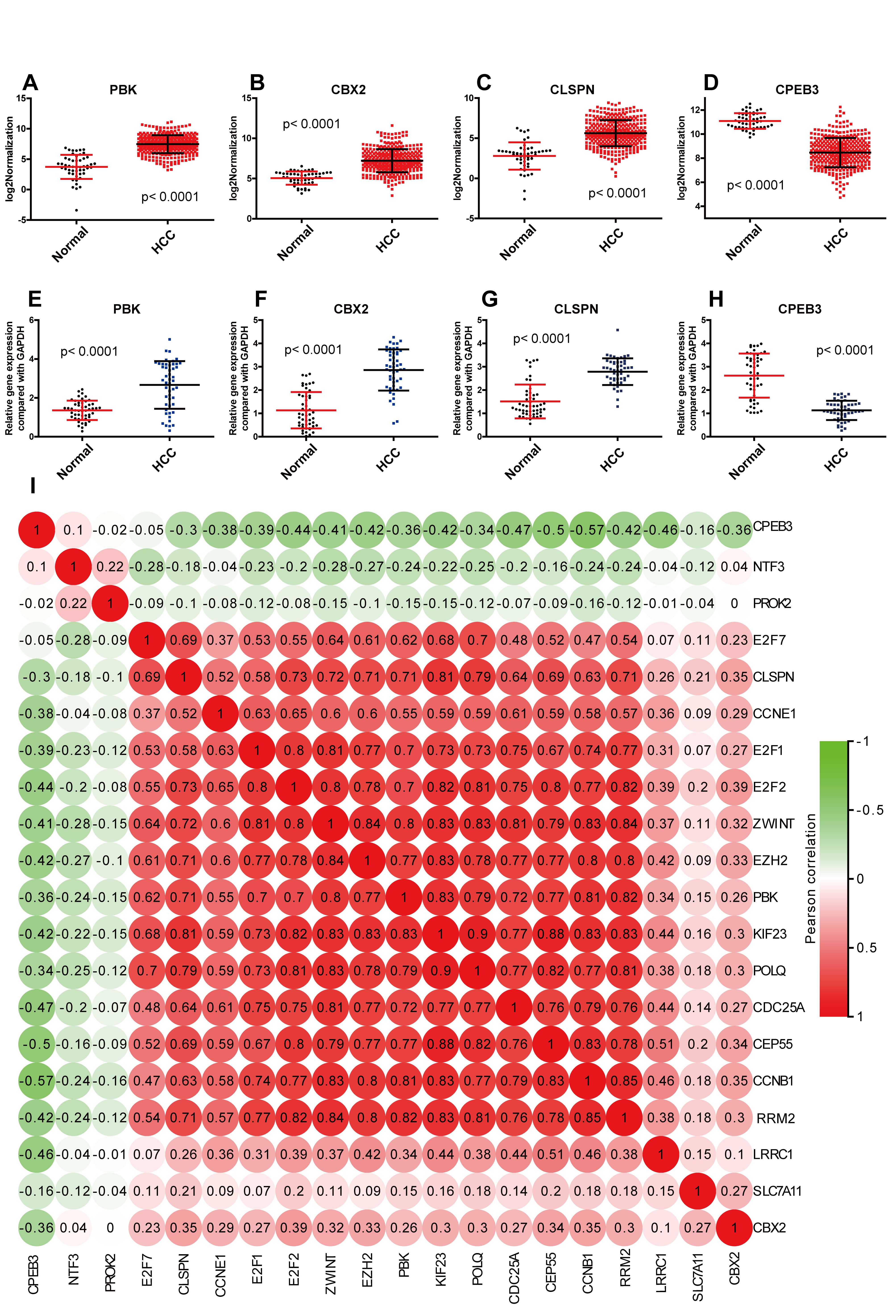

Supplement: Supplementary file 4 — Figure S4 The expression levels of the four genes in the signature and hierarchical clustering in the correlation matrix of 39 DEmRNAs involved in the ceRNA network. The expression levels of the four genes in the prognostic signature in the complete TCGA cohort and SYMH cohort. PBK (A, E), CBX2 (B, F), CLSPN (C, G), and CPEB3 (D, H). Pearson correlation analysis was used to calculate collinearity between the 20 OS-genes in the corresponding row and column (I) (TIFF 7362 kb) [file 12072_2019_9962_MOESM4_ESM.tif]

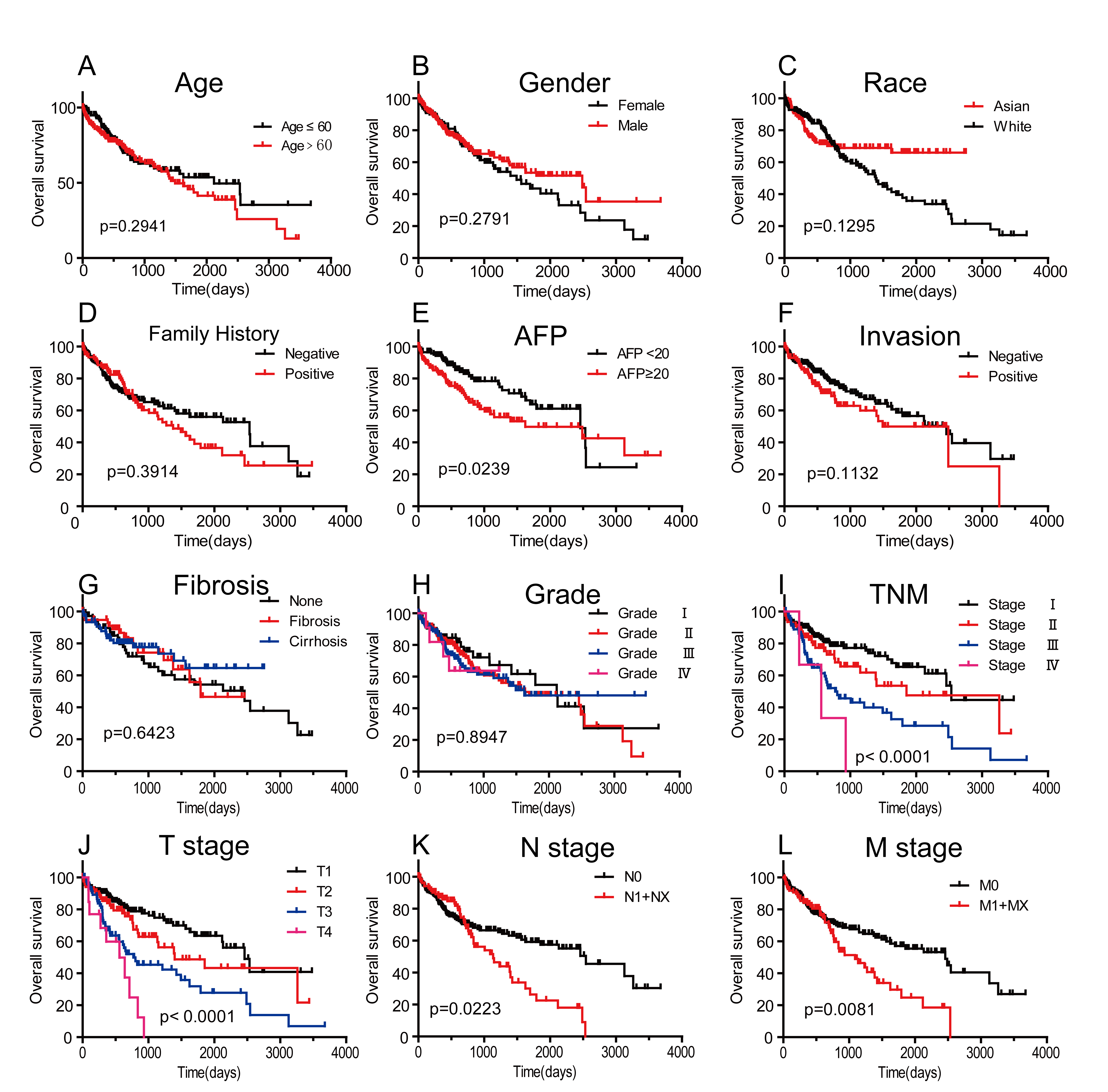

Supplement: Supplementary file 5 — Figure S5 Kaplan–Meier survival curves stratified by age (A), gender (B), race (C), family history (D), serum AFP (E), invasion (F), fibrosis (G), grade (H), TNM (I), T stage (J), N stage (K), and M stage (L) (TIFF 9125 kb) [file 12072_2019_9962_MOESM5_ESM.tif]

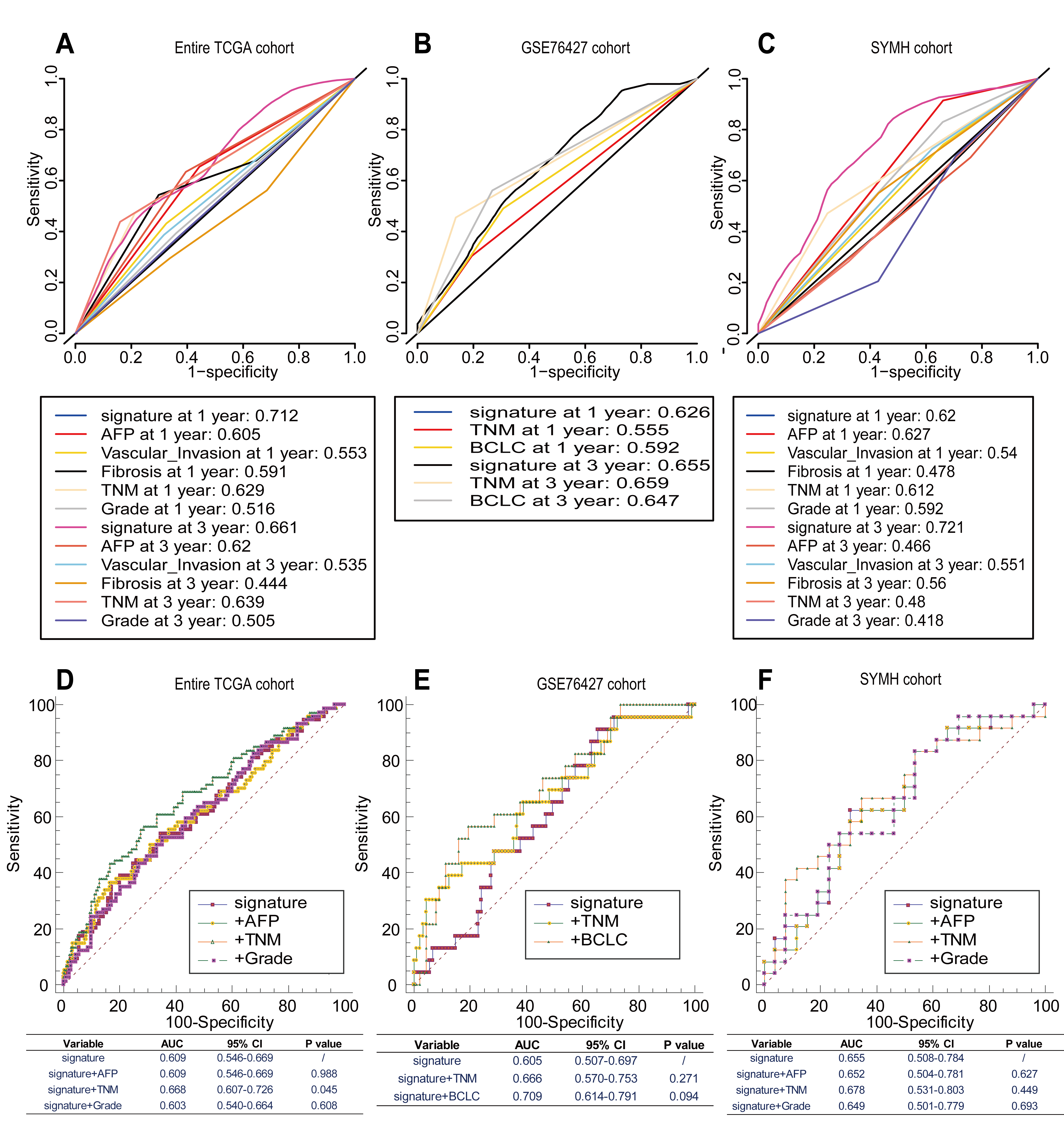

Supplement: Supplementary file 7 — Figure S7 Comparisons of the predictive values for OS of the four-gene-based signature and clinicopathological risk factors according to ROC analysis for 1 and 3 years (A–C). Comparisons of the predictive values for OS of the signature and a combination of the signature and important prognostic factors for independent cohorts (D–F). The AUC was calculated, and its 95% CI was estimated with bootstrap means. The P values were two-sided; HR hazard ratio; 95% CI, 95% confidence interval. (TIFF 10715 kb) [file 12072_2019_9962_MOESM7_ESM.tif]
