# Supplementary material for: Identification and validation of a prognostic four-genes signature for hepatocellular carcinoma: integrated ceRNA network analysis
Source: Hepatol Int. 2019 Jul 18;13(5):618–30. doi: 10.1007/s12072-019-09962-3 (PMC6744548; doi:10.1007/s12072-019-09962-3)
Supplement: Supplementary file 8 — Supplementary material 8 (DOCX 26 kb) [file 12072_2019_9962_MOESM8_ESM.docx]

**Supplementary Table S1 Clinical characteristics for the HCC patients in the TCGA cohort, GSE76427 and SYMH cohort.**

| Clinicopathological Variables | | TCGA Cohort (n=354) | | | *P* Value | External validation cohort | |
| --- | --- | --- | --- | --- | --- | --- | --- |
|  |  | Entire cohort | Training（n=236） | testing（n=118） |  | GSE76427（n=115） | SYMH（n=50） |
| Age | <60 | 161 | 102 | 59 | 0.227 | 48 | 38 |
|  | ≥60 | 193 | 134 | 59 |  | 67 | 12 |
| Gender | Female | 115 | 78 | 37 | 0.748 | 22 | 8 |
|  | male | 239 | 158 | 81 |  | 93 | 42 |
| Race | White | 174 | 116 | 58 | 0.833 | NA | 0 |
|  | Asian | 152 | 103 | 49 |  | NA | 50 |
| Family History | Negative | 198 | 135 | 63 | 0.178 | NA | 42 |
|  | Positive | 109 | 66 | 43 |  | NA | 8 |
| Serum AFP | <20 ng/mL | 142 | 91 | 51 | 0.472 | NA | 14 |
|  | ≥20 ng/mL | 126 | 86 | 40 |  | NA | 36 |
| Vascular invasion | Negative | 198 | 129 | 69 | 0.141 | NA | 16 |
|  | Positive | 102 | 75 | 27 |  | NA | 34 |
| Fibrosis | Negative | 72 | 40 | 32 | 0.339 | NA | 25 |
|  | fibrosis | 67 | 45 | 22 |  | NA | 25 |
|  | Cirrhosis | 67 | 43 | 24 |  | NA |  |
| TNM staging | I-II | 245 | 166 | 79 | 0.759 | 90 | 35 |
|  | III-IV | 85 | 54 | 31 |  | 24 | 15 |
| Tumor grade | I-II | 221 | 150 | 71 | 0.855 | NA | 35 |
|  | III-IV | 128 | 83 | 45 |  | NA | 15 |
| BCLC stage | A | NA | NA | NA | NA | 74 | NA |
|  | B+C | NA | NA | NA |  | 37 | NA |

Abbreviations: SYMH，Sun Yat-Sen Memorial Hospital; AFP, α-fetoprotein; TNM, tumor-lymph node -metastasis; BCLC,

Barcelona Clinic Liver Cancer; OS, overall survival; DFS, disease free survival; NA, not available. Chi-square

test was used for comparison between training set and validation set.

**Supplementary Table S2 Primers for real-time quantitative PCR**

| Gene | sense | antisense |
| --- | --- | --- |
| GAPDH | 5′-GAAGGTGAAGGTCGGAGTCAACG-3′ | 5′-TGCCATGGGTGGAATCATATTGG-3′ |
| PBK | 5′-ATTGGCACAGAGCCATGGAA-3′ | 5′-CCTAGTTCCCAACGCTGCAT-3′ |
| CBX2 | 5′-CTGTGTCAAGGGCAGTGCTA-3′ | 5′-ATACGTGCTCGATGAGGCTG-3′ |
| CLSPN | 5′-CTCACTGCTAAACCAGCCCA-3′ | 5′-ATGCTTCGCGTCAATCCTGA-3′ |
| CPEB3 | 5′-GCACTTAACAATGCCTTCCTGG-3′ | 5′-AGTAGCGTTCTACTCGTTCCC-3′ |

**Supplementary Table S3 20 DEmiRNAs targeting 39 DEmRNAs in ceRNA network.**

| microRNA | Gene |
| --- | --- |
| hsa-mir-96 | PROK2 |
| hsa-mir-93 | ACSL4、KIF23、ELAVL2、NETO2、RRM2、  SALL3、E2F2、EGR2、POLQ、E2F1 |
| hsa-mir-519d | E2F1、ELAVL2、POLQ、NETO2、SALL3、  ACSL4、KIF23、RRM2、E2F2 |
| hsa-mir-506 | ZWINT、LRRC1 |
| hsa-mir-424 | CEP55、HOXA3、E2F7、**CLSPN**、CDC25A、AXIN2、GNAL、KIF23、CCNE1、ITGA2、HOXA10、**CBX2、CPEB3** |
| hsa-mir-373 | **PBK**、SLC7A11、ELAVL2 |
| hsa-mir-372 | SLC7A11、ELAVL2 |
| hsa-mir-31 | HOXC13 |
| hsa-mir-217 | DACH1、EZH2 |
| hsa-mir-21 | NTF3、**CPEB3**、EDIL3 |
| hsa-mir-205 | ACSL4 |
| hsa-mir-183 | GLUL、CCNB1 |
| hsa-mir-182 | NPTX1、HOXA9、FOXF2 |
| hsa-mir-141 | ELAVL2、EPHA2 |
| hsa-mir-137 | PTGS2 |

**Supplementary Table S4 20 DEmiRNAs targeting 83 DElncRNAs in ceRNA network.**

| microRNA | LncRNA |
| --- | --- |
| hsa-mir-137 | LINC00308、TCL6、DSCR8、CLLU1、AC006305.1、AC073263.1、CLDN10-AS1、HTR2A-AS1、AL713998.1、ERVH48-1、GPC6-AS1、CLRN1-AS1、AC012640.1、HOTTIP、RMST |
| hsa-mir-141 | PART1、CCDC13-AS1、AL357153.1、AC024563.1、FAM87A、LINC00308、AL512652.1、CLLU1、AL359878.1、LINC00355、AL713998.1、CCDC26、DLX6-AS1、BPESC1、ERVH48-1、AC009121.1、AC114489.1、HOTTIP、LINC00485 |
| hsa-mir-182 | AP002478.1、LINC00221、TCL6、AC006305.1、AL163952.1、ZNF385D-AS2、LINC00114、SFTA1P、ERVMER61-1、MIR137HG、ERVH48-1、LINC00494、AC012640.1、AC073352.1、GRM5-AS1、RMST、AC040173.1 |
| hsa-mir-183 | C2orf48、AL357153.1、AC024563.1、TCL6、LINC00501、LINC00392、LINC00200、MYCNOS、CRNDE、PVT1、AC040173.1 |
| hsa-mir-184 | AP002478.1、ERVH48-1、HOTTIP、LINC00491 |
| hsa-mir-205 | PART1、CCDC13-AS1、AP002478.1、FAM87A、LINC00308、TCL6、DSCR8、AL512652.1、CLLU1、AL163952.1、LINC00488、LINC00237、ZNF385D-AS1、LINC00351、CCDC26、SACS-AS1、ERVMER61-1、BPESC1、MYCNOS、GPC6-AS1、CLRN1-AS1、AC012640.1、HOTTIP、AC011453.1、CRNDE、PVT1、GRM5-AS1、RMST、LINC00485 |
| **hsa-mir-21** | PART1、AC024563.1、DSCR4、CLLU1、LINC00488、LINC00351、HOTAIR、ERVMER61-1、ERVH48-1、SRGAP3-AS4、GPC6-AS1、PVT1、GRM5-AS1 |
| hsa-mir-216a | C2orf48、AP002478.1、TCL6、AC087392.1、CLLU1、AL359878.1、AC006305.1、LINC00488、AC016773.1、LINC00114、WARS2-IT1、SFTA1P、HOTAIR、LINC00200、DLX6-AS1、BPESC1、CLRN1-AS1、HOTTIP、GDNF-AS1、PVT1、NOVA1-AS1、LINC00485、LINC00519 |
| hsa-mir-216b | FAM87A、DSCR4、TCL6、CLLU1、AL163952.1、LINC00488、WARS2-IT1、SFTA1P、HOTAIR、LINC00200、DLX6-AS1、BPESC1、CLRN1-AS1、AC012640.1、CRNDE、PVT1、LINC00491、GRM5-AS1、NOVA1-AS1 |
| hsa-mir-217 | AL357153.1、AC024563.1、LINC00221、TCL6、CLLU1、AC016773.1、HOTAIR、LINC00200、MIR137HG、MYCNOS、LINC00494、CLRN1-AS1、CRNDE、PVT1、AC040173.1、NOVA1-AS1 |
| hsa-mir-301b | PART1、CCDC13-AS1、AL357153.1、AL033381.1、FAM87A、DSCR4、C17orf82、LINC00221、TCL6、AL512652.1、LINC00501、LINC00272、AL359878.1、SOX21-AS1、HOTAIR、ERVH48-1、AC016912.1、 AL139002.1、HOTTIP、AC068756.1、RMST |
| hsa-mir-31 | PART1、AL357153.1、AL033381.1、FAM87A、DSCR4、LINC00221、TCL6、CLLU1、AP000525.1、AC016773.1、ZNF385D-AS2、LINC00114、ZNF385D-AS1、CCDC26、LINC00200、MIR137HG、DLX6-AS1、LINC00494、HOTTIP、CRNDE、PVT1、GRM5-AS1、RMST、NOVA1-AS1、LINC00485 |
| hsa-mir-372 | C2orf48、AC009065.1、AP002478.1、C10orf91、LINC00221、TCL6、AC087392.1、AC061975.6、CLLU1、AL359878.1、SACS-AS1、DLX6-AS1、LINC00462、LINC00494、LINC00322、HOTTIP、 PVT1、GRM5-AS1、LINC00485 |
| **hsa-mir-373** | C2orf48、AC009065.1、AP002478.1、C10orf91、LINC00221、TCL6、AC087392.1、AC061975.6、CLLU1、AL359878.1、SACS-AS1、DLX6-AS1、LINC00462、LINC00494、LINC00322、HOTTIP、 PVT1、GRM5-AS1、LINC00485 |
| **hsa-mir-424** | PART1、C2orf48、CCDC13-AS1、AL033381.1、AP002478.1、FAM87A、TCL6、AC087392.1、CLLU1、AC006305.1、AC016773.1、LINC00473、WARS2-IT1、SFTA1P、LINC00355、LINC00200、LINC00160、 DLX6-AS1、BPESC1、DSCR10、GPC6-AS1、CLRN1-AS1、HOTTIP、GDNF-AS1、PVT1、RMST、LINC00485 |
| hsa-mir-506 | AL033381.1、FAM87A、LINC00221、LINC00501、AL359878.1、AC006305.1、LINC00355、HOTAIR、LINC00200、SACS-AS1、ERVMER61-1、DLX6-AS1、BPESC1、LINC00316、HOTTIP、PVT1、RMST |
| hsa-mir-508 | PART1、AP002478.1、DSCR4、LINC00221、CLLU1、LINC00488、CLDN10-AS1、DSCR4-IT1、LINC00114、SACS-AS1、BPESC1、ERVH48-1、LINC00494、CRNDE、RMST |
| hsa-mir-519d | C2orf48、AP002478.1、FAM87A、LINC00308、LINC00221、TCL6、AC087392.1、AC061975.6、AL512652.1、TDRG1、CLLU1、AL359878.1、AC006305.1、HOTAIR、LINC00200、DLX6-AS1、 LINC00462、SRGAP3-AS4、HOTTIP、PVT1、GRM5-AS1、RMST、AC040173.1 |
| hsa-mir-93 | C2orf48、AC009065.1、AP002478.1、C10orf91、FAM87A、LINC00308、LINC00221、TCL6、AC087392.1、AC061975.6、AL512652.1、TDRG1、CLLU1、AL359878.1、AC006305.1、HOTAIR、LINC00200、 SACS-AS1、DLX6-AS1、LINC00462、LINC00494、SRGAP3-AS4、LINC00322、HOTTIP、PVT1、GRM5-AS1、RMST、AC040173.1、LINC00485 |
| hsa-mir-96 | FAM87A、LINC00221、TCL6、AL163952.1、LINC00488、ZNF385D-AS2、LINC00114、ERVMER61-1、ERVH48-1、AC073352.1、GRM5-AS1、RMST、AC040173.1 |
| hsa-mir-137 | LINC00308、TCL6、DSCR8、CLLU1、AC006305.1、AC073263.1、CLDN10-AS1、HTR2A-AS1、AL713998.1、ERVH48-1、GPC6-AS1、CLRN1-AS1、AC012640.1、HOTTIP、RMST |

**Supplementary Table S5 The time-dependent ROC curve analysis of the signature**

| Group | 1 year | | 2 years | | | 3 years | | |
| --- | --- | --- | --- | --- | --- | --- | --- | --- |
|  | AUC | 95% CI | AUC | 95% CI | | AUC | 95% CI |  |
| TCGA cohort | 0.712 | 0.636-0.794 | 0.700 | 0.618-0.765 | 0.661 | | 0.594-0.745 |  |
| P value |  | \ |  | 0.032* |  | | <0.01** |  |
| GEO cohort | 0.626 | 0.437-0.752 | 0.632 | 0.489-0.750 | 0.655 | | 0.507-0.750 |  |
| P value |  | \ |  | 0.445 |  | | 0.005** |  |
| SYMH cohort | 0.620 | 0.391-0.768 | 0.637 | 0.473-0.852 | 0.721 | | 0.566-0.871 |  |
| P value |  | \ |  | <0.01** |  | | <0.01** |  |

Abbreviations: SYMH，Sun Yat-Sen Memorial Hospital; 95% CI, 95% confidence interval.

* P<0.05, ** P<0.01.

**Supplementary Table S6 The c-index** **for the 4 genes signature to predict OS**

| Group | OS | |
| --- | --- | --- |
|  | c-index | 95% CI |
| TCGA training set | 0.687 | 0.616-0.757 |
| TCGA validation set | 0.656 | 0.559-0.752 |
| Entire TCGA cohort | 0.674 | 0.617-0.731 |
| GSE76427 cohort | 0.633 | 0.497-0.768 |
| SYMH cohort | 0.672 | 0.543-0.801 |

Abbreviations: SYMH，Sun Yat-Sen Memorial Hospital
